# A Rare Congenital Triad in an Adult With Coronary Artery Disease: A Case Report From Georgia

**DOI:** 10.7759/cureus.92181

**Published:** 2025-09-12

**Authors:** Zviad Bakhutashvili, Lia Janelidze, Nino Lortqipanidze, Konstantine Bliadze, Natia Tamazashvili

**Affiliations:** 1 Department of Cardiothoracic Surgery, Chapidze Emergency Cardiology Center, Tbilisi, GEO; 2 Department of General Surgery, Nassau University Medical Center, East Meadow, USA

**Keywords:** case report, coronary artery bypass grafting, partial anomalous pulmonary venous return, persistent left superior vena cava, sinus venosus asd

## Abstract

A 47-year-old man with a history of New York Heart Association (NYHA) class III heart failure and paroxysmal atrial fibrillation presented with recurrent chest pain, exertional dyspnea, and exercise intolerance. He was diagnosed with an acute myocardial infarction and underwent stenting of the left anterior descending artery. During the same hospitalization, transthoracic echocardiography revealed a sinus venosus atrial septal defect (ASD), moderate tricuspid regurgitation, and right heart dilation, raising suspicion for partial anomalous pulmonary venous drainage (PAPVD). Further imaging confirmed a 1.1 cm sinus venosus ASD with a left-to-right shunt, PAPVD of the right superior pulmonary veins into the superior vena cava (SVC), and a persistent left SVC draining into the coronary sinus. Coronary angiography demonstrated multivessel coronary artery disease.

The patient underwent successful surgical correction, including coronary artery bypass grafting and intracardiac repair with rerouting of anomalous pulmonary veins to the left atrium and ASD closure using an autologous pericardial patch. Postoperative recovery was uneventful aside from transient supraventricular tachycardia with preserved sinus node function and no conduction abnormalities or need for a pacemaker. Two follow-up echocardiographs demonstrated normalization of right heart dimensions, resolution of the shunt, and preserved ejection fraction. This case underscores the importance of considering congenital anomalies in adult patients with new or worsening cardiac symptoms, particularly when incidental findings emerge during evaluation for ischemic heart disease.

## Introduction

Persistent left superior vena cava (PLSVC) is a rare congenital systemic venous anomaly, occurring in approximately 0.3-0.5% of the general population and up to 10% in patients with congenital heart defects [1-3]. Normally, systemic venous return is directed through the inferior vena cava (IVC) and right SVC into the right atrium, while pulmonary veins drain into the left atrium. In our patient, the left-sided cardinal vein persisted, giving PLSVC that drained into the coronary sinus, causing its dilation. In addition, the right superior pulmonary veins drained anomalously into the right SVC, and a superior sinus venosus atrial septal defect (ASD) was also present, giving rise to a left-to-right shunt. It is uncommon to encounter PLSVC in conjunction with a sinus venosus ASD and partial anomalous pulmonary venous drainage (PAPVD) in a single patient. Through an extensive literature review, we found only a handful of cases globally documenting a similar combination of complex congenital heart diseases (CHDs) in adults [1,3,4,5].

This case is particularly significant for several reasons. First, the patient reached adulthood with minimal symptoms, despite having these congenital defects, highlighting the subtle presentation of such anomalies. Second, the coexistence of these congenital anomalies with significant coronary artery disease (CAD) presented a unique surgical challenge, especially given the necessity for coronary artery bypass grafting (CABG). We present this case to highlight the importance of recognizing these complex anomalies in adults, particularly in patients who are candidates for concurrent cardiac interventions.

## Case presentation

In June 2024, a 47-year-old Caucasian man with a known history of NYHA class III heart failure and paroxysmal atrial fibrillation presented with recurrent episodes of anginal chest pain, exertional dyspnea, tachypnea, orthopnea, and severe fatigue unresponsive to his home medications. He was admitted to the ED and was diagnosed with acute MI. He was treated with percutaneous coronary intervention and stenting of the left anterior descending (LAD) artery.

Additional history revealed episodes of dizziness with sudden movements, shortness of breath, and exercise intolerance. During the last two weeks prior to his MI, he had worsening of these symptoms, his exercise tolerance significantly decreased, and he started having nocturnal dyspnea, decreased urination, and lower extremity edema. His past medical history is significant for hypertension, CHF, paroxysmal atrial fibrillation, vertebral trauma, and nephrolithiasis. On physical examination, auscultation revealed a systolic murmur at the left lower sternal border, accentuated with inspiration, as well as a systolic murmur over the pulmonary area. His home medications include aspirin, clopidogrel, nebivolol, rosuvastatin, furosemide, amiodarone, and rivaroxaban. According to him, there was no prior history of any congenital cardiac abnormality.

During this hospitalization, transthoracic echocardiography (TTE) revealed right heart dilation, moderate pulmonary hypertension, moderate tricuspid regurgitation (TR), and a sinus venosus ASD with a left-to-right shunt. PAPVD of the right superior pulmonary vein was suspected. Therefore, CT angiography (CTA) was arranged, and the patient was referred to our center for further evaluation and surgical planning.

His medications after discharge included aspirin, clopidogrel, ramipril, metoprolol, torsemide, spironolactone, rosuvastatin, and pantoprazole.

**Figure 1:**
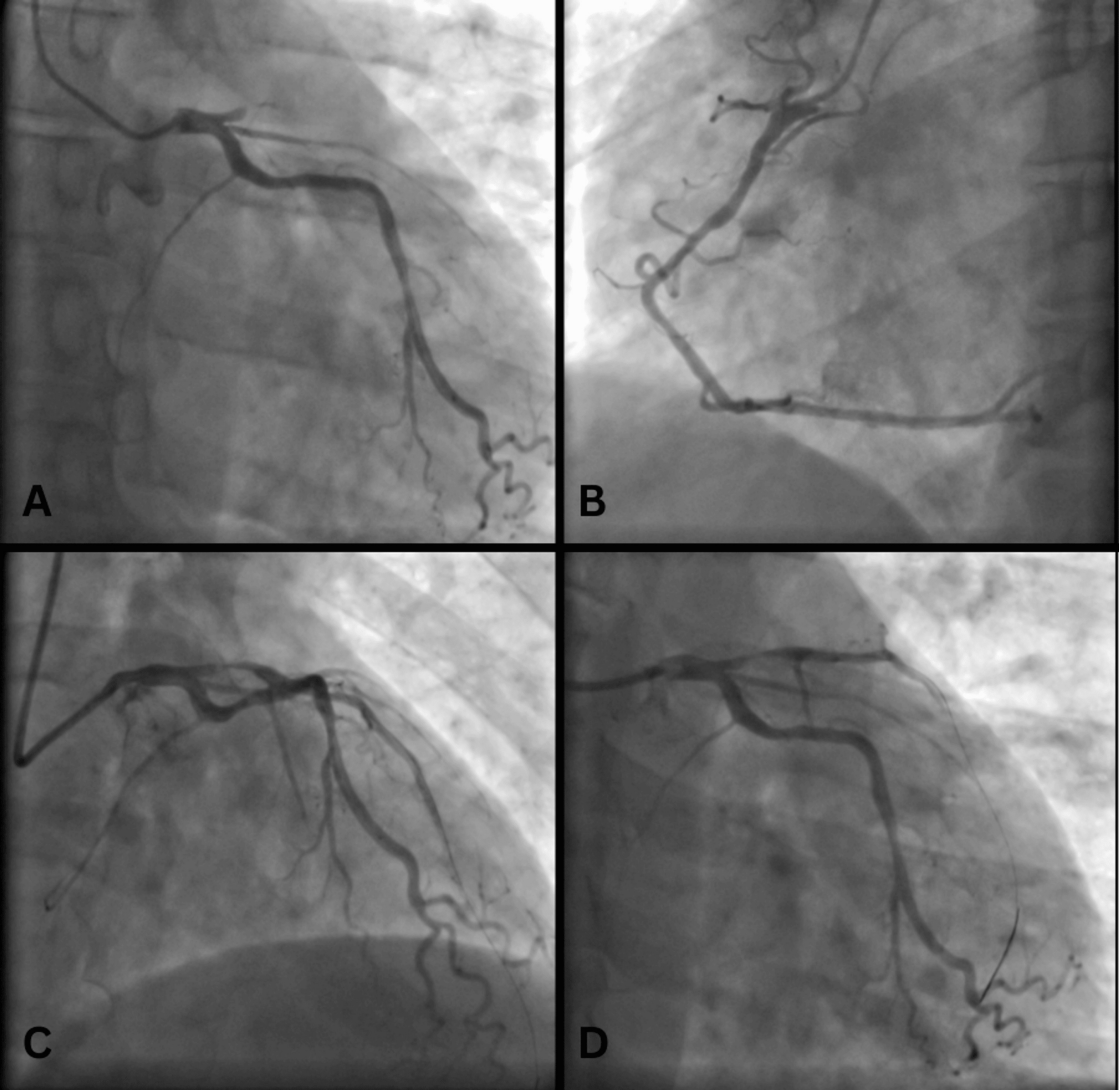
(A) Right anterior oblique (RAO) caudal view of left coronary artery (LCA), LCX and obtuse marginal branches; (B) Left anterior oblique (LAO) caudal view of RCA; (C) RAO cranial view of LAD and LCX; (D) RAO caudal view of LCA, LAD and LCX

**Figure 2:**
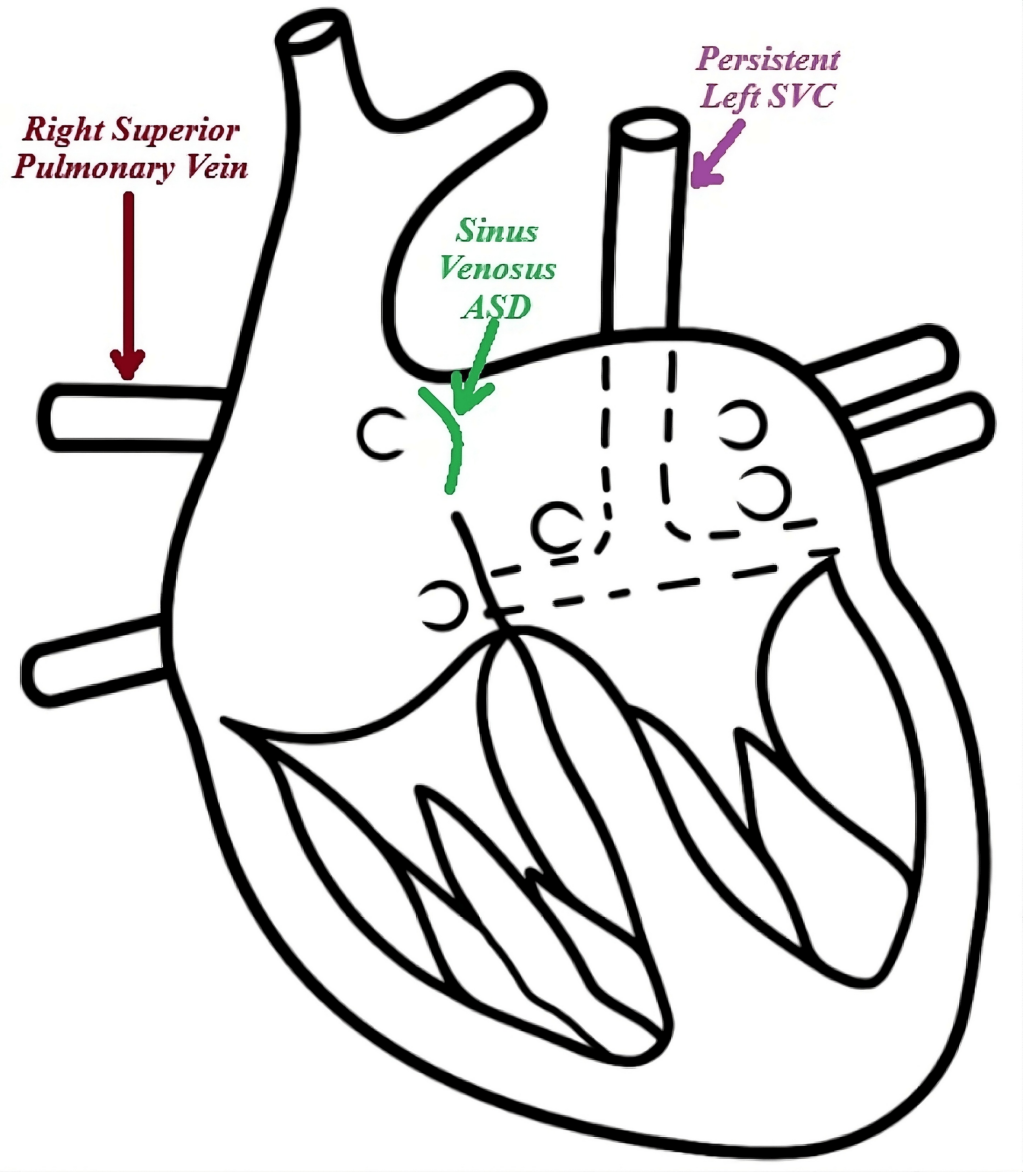
Diagram of congenital abnormalities present in our patient SVC: Superior vena cava; ASD: atrial septal defect

CTA performed in August 2024 confirmed the presence of a 1.1 cm sinus venosus ASD with a left-to-right shunt, PAPVD involving the right superior pulmonary veins draining into the SVC, and persistent left SVC draining into the coronary sinus. Coronary angiography showed <50% stenosis in the right coronary artery (RCA), the LAD stent with >50% in-stent stenosis, <50% stenosis in the mid-LAD, and >70% diffuse stenosis in the left circumflex artery (LCX) (Figure 1). The patient's logistic EuroSCORE was 6.41%. A schematic diagram illustrating these congenital abnormalities is shown in Figure 2.

Repeat preoperative TTE in September 2024 showed left ventricular ejection fraction (LVEF) of 40%, global systolic dysfunction, mild mitral regurgitation (MR) due to restricted posterior leaflet motion, moderate TR with a vena contracta of 0.55 cm, and akinesia of the interventricular septum (IVS). It also confirmed CTA findings and showed right atrial (RA) and right ventricular (RV) dilation (5.1 cm and 4.9 cm, respectively) with a pulmonary artery systolic pressure (PASP) of 38 mm Hg.

During follow-up, the patient complained of persistent shortness of breath and anginal chest pain despite optimal medical therapy. Given the ongoing symptoms, presence of multivessel CAD, and the need for concurrent correction of congenital defects, as well as potential consequences of them being uncorrected, CABG with correction of concurrent anomalies was planned in September 2024.

Surgical intervention

**Figure 3:**
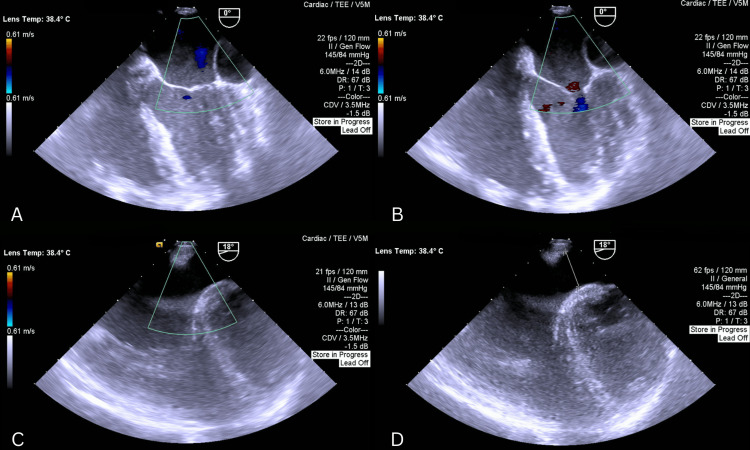
(A, B) Four-chamber views demonstrating a markedly dilated coronary sinus, along with an enlarged left atrium and left ventricle; the mitral valve is visualized; (C, D) Modified views demonstrating the atrial septal defect (ASD)

**Figure 4:**
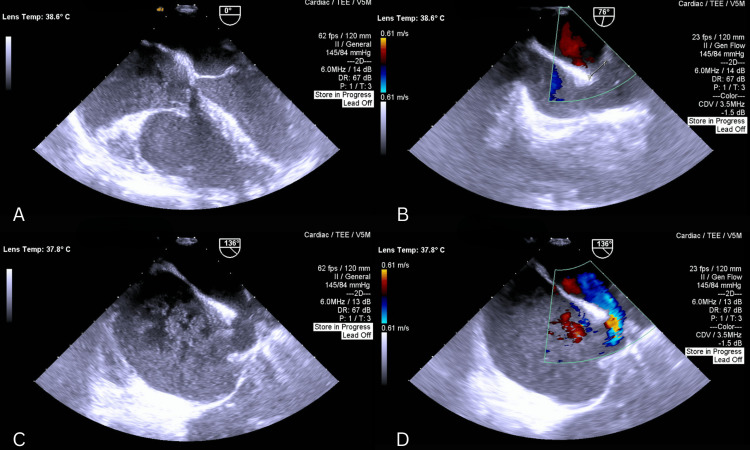
(A) Four-chamber view demonstrating enlarged RA and RV; TV is visualized; (B, C, D) Bicaval views showing the sinus venosus atrial septal defect (ASD) with left-to-right shunt on color Doppler (D)

The surgery was performed through a median sternotomy with a total operative time of 210 minutes, a cardiopulmonary bypass (CPB) time of 110 minutes, and an aortic cross-clamp time of 80 minutes. Preoperative transesophageal echocardiogram (TEE) reconfirmed the presence of the above-mentioned defects with a coronary sinus diameter measuring 2.2 cm (Figure 3, Figure 4).

The pericardium was opened, and stay sutures were applied. Purse-string sutures were placed in the ascending aorta for the aortic and cardioplegic cannulae, and similar sutures were placed in both SVC and inferior vena cava (IVC) for cannulation. CPB was established, and all these cannulas were fixed with tourniquets. A left atrial vent was inserted through the mitral valve into the left ventricle. The distal anastomosis for CABG was performed between the left internal mammary artery (LIMA) and the LAD, as well as between the great saphenous vein (GSV) and the LCX.

Following distal anastomosis, the SVC and IVC were clamped, and the RA was opened through the usual oblique incision. Openings of the right pulmonary veins into the SVC were identified. Using an autologous pericardial patch, the sinus venosus ASD was closed, and the pulmonary veins were baffled toward the left atrium (LA), allowing normal pulmonary venous drainage. The RA was closed with 5-0 Prolene sutures, and proximal anastomoses of the CABG grafts were completed.

**Figure 5:**
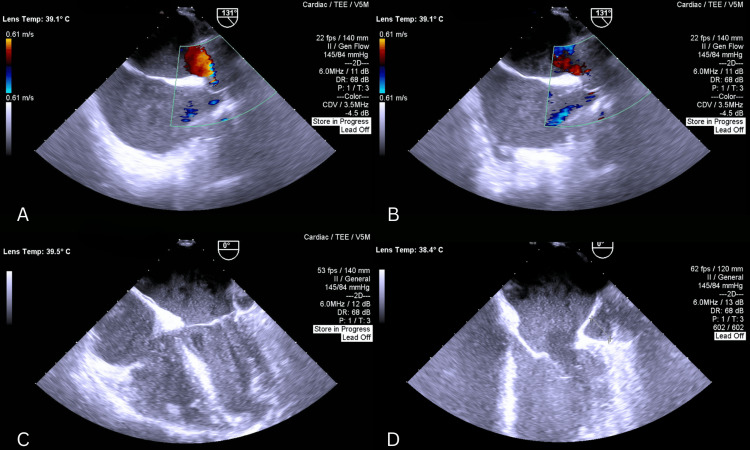
(A, B) Bicaval views with Doppler demonstrating closed ASD with no residual flow across the septum. (C) Four-chamber view showing a reduced right ventricular size and a dilated coronary sinus. (D) Measurement of dilated coronary sinus diameter ASD: Atrial septal defect

Postoperative TEE confirmed successful closure of the ASD, reduced RA and RV dimensions, and correction of PAPVD, though coronary sinus dilation persisted (Figure 5). The patient was transferred to the ICU, where he underwent the standard postoperative course without any significant complications.

At the follow-up appointment in October 2024, TTE showed reduced RA and RV diameters (3.6 cm each), improved LVEF to 51%, and no residual ASD or shunt. Mild MR and TR persisted, and PASP was reduced to 26 mm Hg. These findings indicate improved right-sided dimensions and overall ventricular function, with only mild residual valvular regurgitation. At six-month follow-up, the patient remained clinically asymptomatic and his TTE findings remained the same except mild diastolic dysfunction and myocardial hypertrophy with scar formation in the anterior septal and apical walls, which was consistent with prior MI. He remained on aspirin, clopidogrel, ramipril, metoprolol, torsemide, spironolactone, rosuvastatin, and pantoprazole.

Written informed consent was obtained from the patient for publication of this case report and accompanying images.

## Discussion

Congenital heart defects are becoming increasingly prevalent among adults, with an estimated 3,500 per million affected due to advancements in diagnosis, ICU care, and surgical techniques available now for children with CHDs [6,7]. Despite this rise, persistent left SVC remains rare in the general population, occurring in under 0.5% [1-3]. PLSVC is caused by the failure of the caudal left anterior cardinal vein to regress after eight weeks of embryonic development [8]. It may coincide with other defects, including ASD [1]. Different subtypes of ASD, deficiency of the interatrial septum, include ostium primum defect, ostium secundum defect, patent foramen ovale, sinus venosus ASD, or coronary sinus defect [9]. In our patient, PLSVC was accompanied by sinus venosus ASD and abnormal pulmonary venous drainage. As mentioned above, we were able to find only a few similar cases (Table 1) [1,3,4,5].

**Table 1:** Rapid review of case reports with similar congenital abnormalities

	Age/ Sex	Anatomical Abnormality	History and Physical Examination	ECG	Hemodynamics	Repair Type	Concomitant Surgery	Concomitant Abnormality	Outcome
Case 1 [1]	57/F	PLSVC draining into dilated coronary sinus; Anomalous drainage of right superior pulmonary vein into R SVC; Sinus venosus ASD	Asymptomatic; Systolo-diastolic murmur over mitral and aortic areas, 2/6 pansystolic murmur over the tricuspid area	Sinus rhythm, incomplete Right Bundle Branch Block (RBBB)	Biatrial and RV dilatation; Enlarged main pulmonary artery and coronary sinus; Moderate TR; Pulmonary hypertension (PAP = 50 mmHg); LVEF - 64%; Pulmonary to systemic flow ratio (QP/QS) - 2.34	Surgery was suggested, but declined by the patient	NA	NA	
Case 2 [3]	47F	PLSVC draining into dilated coronary sinus; Anomalous drainage of right superior pulmonary vein into R SVC; No intracardiac shunt	Worsening anginal chest pain and shortness of breath; Systolic ejection murmur at the left sternal border; 70-80% occlusion of LAD; 90% occlusion of LCX; 90% occlusion of RCA	Sinus rhythm, 1^st^ degree AV block, LA enlargement, incomplete RBBB	LV hypertrophy; Mild RA and RV enlargement; Mild aortic regurgitation (AR) and aortic stenosis (AS); Mild MR; Mild pulmonary regurgitation (PR)	Intracardiac baffle with Gore-Tex	CABG	NA	Died from septic shock a few weeks after surgery; Source – incision site at the thigh
Case 3 [4]	40F	PLSVC draining into dilated coronary sinus; Anomalous drainage of right pulmonary veins into small R SVC; Sinus venosus ASD (known)	Exertional dyspnea and fatigue; Fixed splitting of S2 and systolic murmur above the pulmonary valve	1^st^ degree AV block	Enlarged RV and both atria; Pulmonary to systemic flow ratio – 3.7:1	ASD widened and closed by autologous pericardial patch	NA	Left-sided IVC draining into PLSVC; Direct drainage of hepatic veins in RA	
Case 4 [5]	57/F	PLSVC draining into nondilated coronary sinus; Anomalous drainage of the right superior pulmonary vein into small R SVC; No intracardiac shunt	Dyspnea on exertion for four days, paroxysmal nocturnal dyspnea, orthopnea, difficulty completing a full sentence, inability to walk more than few feet, hypertension; 3/6 crescendo-decrescendo murmur with radiation to the right carotid, bibasilar crackles, pitting edema in the bilateral lower extremities	Normal sinus rhythm	LVEF - 55%; Moderate AS; Peak velocity 3.66 m/s; Mean gradient 34 mmHg	No surgical repair	NA	NA	Asymptomatic with medical management for two years

What makes this case particularly noteworthy is that the PLSVC and associated defects were undiagnosed for 47 years, during which the patient remained largely asymptomatic. This highlights the potential for long-term survival in individuals with these defects, often leading to late diagnosis and discovery only during imaging for unrelated issues. Our patient was initially assessed for complications related to myocardial infarction (MI), illustrating how such anomalies can go unnoticed for decades. This combination of defects is rarely reported in adults; therefore, it adds significant value to the literature; to our knowledge, no prior cases from Georgia have been published.

Patients with PLSVC are often asymptomatic and found incidentally during imaging or procedures [1,2]. This was true for our patient, who was initially assessed for complications of MI. Some patients may even seek care due to associated CHDs or arrhythmias associated with PLSVC [2,5].

Diagnosis typically involves echocardiography [2], as with our patient, where TTE and TEE revealed a dilated coronary sinus, in addition to other abnormalities. For detailed characterization and surgical planning, CT angiography or MRI is invaluable [1-4]. In our case, CTA confirmed the ASD, identified PAPVD, identified double SVC, and ruled out additional defects, proving useful in this adult patient with concurrent CAD.

Identification of left SVC preoperatively is vital when CPB is required for two reasons: 1) Venous cannulation of the RA might be inadequate to drain the blood into the venous reservoir, and 2) retrograde cardioplegia into the coronary sinus may pose challenges [3]. Additionally, identifying left SVC prevents complications associated with left subclavian catheterization in this group of patients [2].

Based on the REVEAL Registry data, the long-term complications of uncorrected CHD are significant and closely linked to the development of pulmonary arterial hypertension, particularly in its most severe form - Eisenmenger syndrome (ES) [10]. ES leads to irreversible pulmonary vascular remodeling, resulting in right-to-left shunting, severe hypoxemia, and inoperable cardiac lesions [10]. Patients with uncorrected CHD who progress to ES exhibit more derangement in echocardiographic parameters, persistent symptoms and are more likely to experience pericardial effusion, impaired exercise capacity, and right ventricular dysfunction [10]. Predicted 10-year mortality in the patients with uncorrected CHDs ranges from 13.7 to around 53%, based on the complexity of CHD [11].

This underscores that, even if CAD alone wasn't an absolute indication for surgery, the presence of uncorrected structural heart defects significantly elevated long-term risk, justifying a more aggressive surgical approach.

## Conclusions

In summary, this case presents a rare combination of a persistent left SVC, sinus venosus ASD, and PAPVD in an adult patient presenting with concurrent multivessel CAD. The fact that these abnormalities remained undetected for decades highlights how subtle their presentation is and how important it is to take congenital heart defects into consideration in adults, especially when they come with unexplained symptoms. This case emphasizes how important careful surgical planning and comprehensive preoperative imaging are, especially in patients with complex congenital heart defects. To minimize potential problems during CPB and to maximize surgical results, early detection of PLSVC and related abnormalities is important. Timely surgical correction of these defects is essential to prevent long-term complications such as right heart failure, pulmonary hypertension, arrhythmias, and irreversible vascular remodeling. This case contributes valuable insights to the limited literature on complex adult CHD and reinforces the importance of considering congenital defects in adult patients with unexplained cardiac symptoms.
